# 2-Methyl­benzaldehyde 2-methyl­benzyl­idenehydrazone

**DOI:** 10.1107/S160053680801934X

**Published:** 2008-07-05

**Authors:** Shang Shan, Wen-Long Wang, Pei-Jin Xie, Ying-Li Xu, Shan-Heng Wang

**Affiliations:** a College of Chemical Engineering and Materials Science, Zhejiang University of Technology, People’s Republic of China

## Abstract

The mol­ecule of the title compound, C_16_H_16_N_2_, is centrosymmetric and the dihedral angle between the benzene ring and the dimethyl­hydrazine mean plane is 16.11 (15)°.

## Related literature

For background, see: Shan *et al.* (2003 ▶). For related structures, see: Fan *et al.* (2008 ▶); Shan *et al.* (2004 ▶, 2008 ▶).
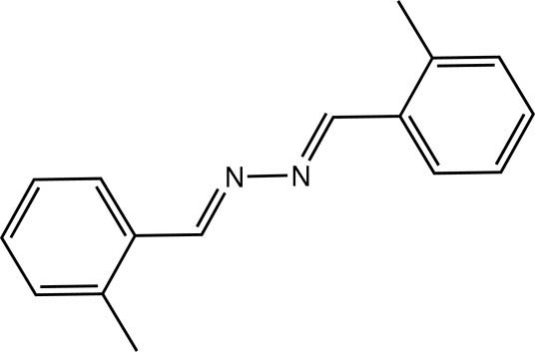


## Experimental

### 

#### Crystal data

C_16_H_16_N_2_
*M* *_r_* = 236.31
Monoclinic, 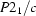

*a* = 6.1578 (11) Å
*b* = 13.248 (2) Å
*c* = 8.8161 (16) Å
β = 105.398 (12)°
*V* = 693.4 (2) Å^3^
*Z* = 2
Mo *K*α radiation
μ = 0.07 mm^−1^
*T* = 295 (2) K
0.32 × 0.28 × 0.12 mm

#### Data collection

Rigaku R-AXIS RAPID IP diffractometer
Absorption correction: none
5451 measured reflections
1503 independent reflections
1168 reflections with *I* > 2σ(*I*)
*R*_int_ = 0.020

#### Refinement

*R*[*F* ^2^ > 2σ(*F* ^2^)] = 0.038
*wR*(*F* ^2^) = 0.115
*S* = 1.10
1503 reflections
84 parameters
H-atom parameters constrained
Δρ_max_ = 0.12 e Å^−3^
Δρ_min_ = −0.10 e Å^−3^

### 

Data collection: *PROCESS-AUTO* (Rigaku, 1998 ▶); cell refinement: *PROCESS-AUTO*; data reduction: *CrystalStructure* (Rigaku/MSC, 2002 ▶); program(s) used to solve structure: *SIR92* (Altomare *et al.*, 1993 ▶); program(s) used to refine structure: *SHELXL97* (Sheldrick, 2008 ▶); molecular graphics: *ORTEP-3 for Windows* (Farrugia, 1997 ▶); software used to prepare material for publication: *WinGX* (Farrugia, 1999 ▶).

## Supplementary Material

Crystal structure: contains datablocks I, global. DOI: 10.1107/S160053680801934X/hb2750sup1.cif

Structure factors: contains datablocks I. DOI: 10.1107/S160053680801934X/hb2750Isup2.hkl

Additional supplementary materials:  crystallographic information; 3D view; checkCIF report

## Figures and Tables

**Fig. 1.**
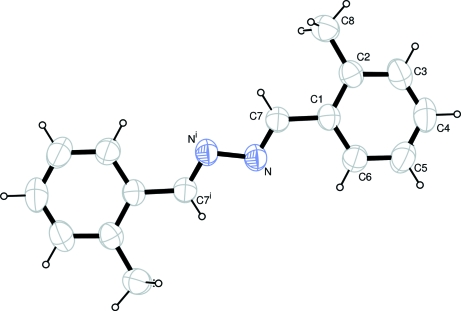
The molecular structure of (I) with 50% probability displacement ellipsoids for non-H atoms. Symmetry code: (i) -x, 1-y, -z.

## supplementary crystallographic information

### Comment

As part of our ongong studies of hydrazone derivatives (Shan *et al.*, 2003), the title compound, (I), has been prepared and its crystal structure is reported here (Fig. 1).

The molecule of (I) is centrosymmetric, with the mid-point of the N—N bond located on an inversion center. The N=C7 double bond distance of 1.2727 (14) Å is shorter than the C=N bond distances found in related hydrazone structures, i.e. 1.295 (2)Å in (*E*)-3-methoxyacetophenone 4-nitrophenylhydrazone (Fan *et al.*, 2008), 1.2977 (18) Å in (E)-2-furyl methyl ketone 2,4-dinitrophenylhydrazone (Shan *et al.*, 2008) and 1.293 (2) Å in benzylideneacetone 2,4-dinitrophenylhydrazone (Shan *et al.* 2004). In (I), the terminal benzene ring is twisted with respect to the central dimethylhydrazine plane by 16.11 (15)°.  The crystal packing is controlled by van der Waals forces.

### Experimental

Hydrazine hydrate (0.10 g, 2 mmol) was dissolved in ethanol (10 ml), then acetic acid (0.1 ml) was added slowly to the ethanol solution with stirring. The solution was heated at 333 K for several minutes until the solution cleared. 2-Methylbenzaldehyde (0.24 g, 2 mmol) was then dropped slowly into the solution, and the mixture was kept at 333 K with continuous stirring for 2 h. After the solution had cooled to room temperature yellow powder appeared. The crude title compound was separated and washed with water three times. Recrystallization from an absolute ethanol yielded yellow plates of (I).

### Refinement

Methyl H atoms were placed in calculated positions with C—H = 0.96 Å and the torsion angle was refined to fit the electron density with *U*_iso_(H) = 1.5*U*_eq_(C). The other H atoms were placed in calculated positions with C—H = 0.93 and refined as riding with *U*_iso_(H) = 1.2*U*_eq_(C).

### Crystal data

**Table:**

C_16_H_16_N_2_	*F*_000_ = 252
*M**_r_* = 236.31	*D*_x_ = 1.132 Mg m^−^^3^
Monoclinic, *P*2_1_/*c*	Mo *K*α radiation λ = 0.71073 Å
Hall symbol: -P 2ybc	Cell parameters from 2246 reflections
*a* = 6.1578 (11) Å	θ = 3.0–25.5º
*b* = 13.248 (2) Å	µ = 0.07 mm^−^^1^
*c* = 8.8161 (16) Å	*T* = 295 (2) K
β = 105.398 (12)º	Plate, yellow
*V* = 693.4 (2) Å^3^	0.32 × 0.28 × 0.12 mm
*Z* = 2	

### Data collection

**Table:**

Rigaku R-AXIS RAPID IP diffractometer	1503 independent reflections
Radiation source: fine-focus sealed tube	1168 reflections with *I* > 2σ(*I*)
Monochromator: graphite	*R*_int_ = 0.020
Detector resolution: 10.00 pixels mm^-1^	θ_max_ = 27.0º
*T* = 295(2) K	θ_min_ = 2.9º
ω scans	*h* = −7→7
Absorption correction: none	*k* = −15→16
5451 measured reflections	*l* = −11→11

### Refinement

**Table:**

Refinement on *F*^2^	Hydrogen site location: inferred from neighbouring sites
Least-squares matrix: full	H-atom parameters constrained
*R*[*F*^2^ > 2σ(*F*^2^)] = 0.039	*w* = 1/[σ^2^(*F*_o_^2^) + (0.0537*P*)^2^ + 0.0481*P*] where *P* = (*F*_o_^2^ + 2*F*_c_^2^)/3
*wR*(*F*^2^) = 0.115	(Δ/σ)_max_ < 0.001
*S* = 1.10	Δρ_max_ = 0.12 e Å^−^^3^
1503 reflections	Δρ_min_ = −0.10 e Å^−^^3^
84 parameters	Extinction correction: SHELXL97 (Sheldrick, 2008), Fc^*^=kFc[1+0.001xFc^2^λ^3^/sin(2θ)]^-1/4^
Primary atom site location: structure-invariant direct methods	Extinction coefficient: 0.099 (12)
Secondary atom site location: difference Fourier map	

### Special details

**Table:**

Geometry. All e.s.d.'s (except the e.s.d. in the dihedral angle between two l.s. planes) are estimated using the full covariance matrix. The cell e.s.d.'s are taken into account individually in the estimation of e.s.d.'s in distances, angles and torsion angles; correlations between e.s.d.'s in cell parameters are only used when they are defined by crystal symmetry. An approximate (isotropic) treatment of cell e.s.d.'s is used for estimating e.s.d.'s involving l.s. planes.
Refinement. Refinement of *F*^2^ against ALL reflections. The weighted *R*-factor *wR* and goodness of fit *S* are based on *F*^2^, conventional *R*-factors *R* are based on *F*, with *F* set to zero for negative *F*^2^. The threshold expression of *F*^2^ > σ(*F*^2^) is used only for calculating *R*-factors(gt) *etc*. and is not relevant to the choice of reflections for refinement. *R*-factors based on *F*^2^ are statistically about twice as large as those based on *F*, and *R*- factors based on ALL data will be even larger.

### Fractional atomic coordinates and isotropic or equivalent isotropic displacement parameters (Å^2^)

**Table:**

	*x*	*y*	*z*	*U*_iso_*/*U*_eq_	
N	0.03959 (16)	0.45944 (7)	0.05193 (11)	0.0545 (3)	
C1	0.33920 (18)	0.40583 (8)	0.26999 (12)	0.0495 (3)	
C2	0.56653 (19)	0.41758 (9)	0.35123 (13)	0.0553 (3)	
C3	0.6648 (2)	0.34407 (11)	0.46155 (15)	0.0731 (4)	
H3	0.8162	0.3499	0.5151	0.088*	
C4	0.5435 (3)	0.26319 (11)	0.49318 (18)	0.0813 (5)	
H4	0.6129	0.2158	0.5682	0.098*	
C5	0.3198 (3)	0.25229 (10)	0.41412 (16)	0.0754 (4)	
H5	0.2377	0.1977	0.4356	0.090*	
C6	0.2186 (2)	0.32277 (9)	0.30309 (14)	0.0617 (4)	
H6	0.0678	0.3151	0.2492	0.074*	
C7	0.22666 (19)	0.47938 (8)	0.15044 (13)	0.0514 (3)	
H7	0.2935	0.5419	0.1466	0.062*	
C8	0.7057 (2)	0.50502 (11)	0.32086 (16)	0.0720 (4)	
H8A	0.6477	0.5668	0.3517	0.108*	
H8B	0.6986	0.5078	0.2108	0.108*	
H8C	0.8593	0.4963	0.3807	0.108*	

### Atomic displacement parameters (Å^2^)

**Table:**

	*U*^11^	*U*^22^	*U*^33^	*U*^12^	*U*^13^	*U*^23^
N	0.0577 (6)	0.0502 (6)	0.0522 (6)	0.0060 (4)	0.0086 (4)	0.0054 (4)
C1	0.0564 (6)	0.0494 (6)	0.0427 (6)	0.0049 (5)	0.0129 (5)	−0.0008 (5)
C2	0.0563 (7)	0.0624 (7)	0.0471 (6)	0.0058 (5)	0.0137 (5)	−0.0003 (5)
C3	0.0655 (8)	0.0849 (10)	0.0623 (8)	0.0139 (7)	0.0055 (6)	0.0119 (7)
C4	0.0945 (11)	0.0741 (9)	0.0686 (9)	0.0164 (8)	0.0097 (8)	0.0235 (7)
C5	0.0976 (11)	0.0571 (8)	0.0700 (9)	−0.0035 (7)	0.0198 (8)	0.0121 (6)
C6	0.0682 (8)	0.0567 (7)	0.0570 (7)	−0.0038 (6)	0.0112 (6)	0.0030 (5)
C7	0.0549 (6)	0.0492 (6)	0.0495 (6)	0.0012 (5)	0.0126 (5)	0.0009 (5)
C8	0.0568 (7)	0.0860 (10)	0.0711 (8)	−0.0059 (6)	0.0135 (6)	0.0053 (7)

### Geometric parameters (Å, °)

**Table:**

N—C7	1.2727 (14)	C4—C5	1.376 (2)
N—N^i^	1.4121 (17)	C4—H4	0.9300
C1—C6	1.4007 (16)	C5—C6	1.3764 (17)
C1—C2	1.4016 (16)	C5—H5	0.9300
C1—C7	1.4672 (15)	C6—H6	0.9300
C2—C3	1.3956 (17)	C7—H7	0.9300
C2—C8	1.5065 (17)	C8—H8A	0.9600
C3—C4	1.3761 (19)	C8—H8B	0.9600
C3—H3	0.9300	C8—H8C	0.9600
			
C7—N—N^i^	112.25 (11)	C4—C5—H5	120.3
C6—C1—C2	119.57 (10)	C6—C5—H5	120.3
C6—C1—C7	119.74 (10)	C5—C6—C1	121.08 (12)
C2—C1—C7	120.70 (10)	C5—C6—H6	119.5
C3—C2—C1	117.90 (12)	C1—C6—H6	119.5
C3—C2—C8	119.92 (11)	N—C7—C1	121.44 (11)
C1—C2—C8	122.18 (10)	N—C7—H7	119.3
C4—C3—C2	121.77 (13)	C1—C7—H7	119.3
C4—C3—H3	119.1	C2—C8—H8A	109.5
C2—C3—H3	119.1	C2—C8—H8B	109.5
C5—C4—C3	120.20 (12)	H8A—C8—H8B	109.5
C5—C4—H4	119.9	C2—C8—H8C	109.5
C3—C4—H4	119.9	H8A—C8—H8C	109.5
C4—C5—C6	119.48 (13)	H8B—C8—H8C	109.5

Symmetry codes: (i) −*x*, −*y*+1, −*z*.
